# Effect of Zusanli Acupoint Injection with Anisodamine on Postoperative Recovery Quality of Patients Undergoing Bariatric Surgery

**DOI:** 10.1007/s11695-024-07182-9

**Published:** 2024-03-20

**Authors:** Jianxin Cheng, Xiaohan Wang, Rui Wang, Jingyi Sheng, Shanshan Guo, Tianya Liu, Zhiping Wang

**Affiliations:** Department of Anesthesiology, The Affiliated Hospital of Xuzhou Medical University, Xuzhou, 221002 China

**Keywords:** ST36, Acupoint injection, Anisodamine, Laparoscopic sleeve gastrectomy, Postoperative recovery quality

## Abstract

**Purpose:**

To evaluate the influence of anisodamine injection at the Zusanli (ST36) on early postoperative recovery quality in patients who have undergone laparoscopic sleeve gastrectomy.

**Materials and Methods:**

141 patients undergoing laparoscopic sleeve gastrectomy were randomly divided into the control group (group C), the normal saline group (group S) and the anisodamine group (group A). Acupuncture point injections were administered after induction of general anesthesia. The quality of recovery-40 questionnaire (QoR-40) scores were documented preoperatively (D0) and on the 1st (D1), 3rd (D3) and 7th (D7) days postoperatively. Additional metrics included: the numerical rating scale (NRS) for pain, postoperative nausea and vomiting (PONV), assessment and analgesic consumption 24-h post-extubation and the initial postoperative times for ambulation and anal exhaust. Substance P (SP), β-endorphin (β-EP), motilin (MTL) and gastrin (GAS) were quantified at 24-h post-surgery.

**Results:**

Compared with group C, group A demonstrated an elevation in QoR-40 scores and physical comfort dimensions during D1-3, and an increased pain scores during D1-7; group S exhibited an augmentation in QoR-40 scores and pain scores on D1 (*p* < 0.05). Compared with group S, group A improved QoR-40 scores on D1 and pain scores during D1-3 (*p* < 0.05). SP, β-EP, MTL and GAS presented significant variances among the groups 24-h post-surgery (*p* < 0.05). There were significant differences between the groups in NRS pain scores and PONV scores at 24-h postoperatively, dosage of dizocin on the first postoperative day, and time to first anal defecation (*p* < 0.05).

**Conclusion:**

The administration of anisodamine via ST36 acupoint injections has been demonstrated to facilitate the recuperation of gastrointestinal functionality, to alleviate postoperative pain and nausea, and substantially to enhance the quality of early postoperative recovery.

**Graphical Abstract:**

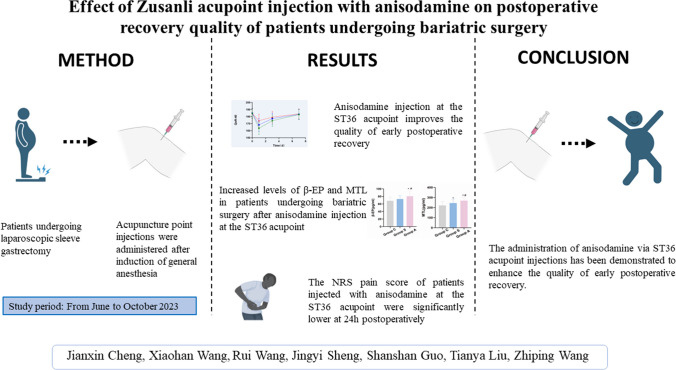

## Background

The escalating prevalence of obesity is an alarming trend, with nearly 20% of minors and over 50% of adults currently grappling with overweight and obesity-related issues in China, thereby posing a significant public health challenge [1]. For individuals with severe obesity, conventional weight-loss strategies such as caloric restriction and physical exercise often fall short in adherence and effectiveness, positioning bariatric surgery as the sole long-term efficacious intervention [2]. Moreover, clinical evidence has underscored the efficacy of bariatric surgery in not only mitigating obesity and overweight conditions but also ameliorating related comorbidities, including type 2 diabetes, hypertension and hyperlipidemia [3–5]. Nevertheless, the perioperative period for bariatric surgery patients is marred by numerous adverse events such as infections, pain, nausea, vomiting and intestinal obstructions, which profoundly impede wound healing and gastrointestinal function recovery [6, 7]. Hence, enhancing the overall anesthetic management and fostering expedited patient recovery constitute pivotal challenges and focal points in perioperative bariatric surgery care [8]. In recent years, ST36 acupoint injection of anisodamine has received increasing attention as a new therapeutic approach [9]. Studies have shown that ST36 acupoint injection of anisodamine has a good effect in improving PONV and other clinical adverse symptoms [10]. Therefore, the purpose of this study was to explore the effect of acupoint injection of anisodamine at ST36 on the quality of early recovery after bariatric surgery, in order to provide reference for clinical practice.

## Methods

### Study Design

Based on the preliminary test results, the mean QoR-40 scores at 24 h post-operation in group A, S and C were 174, 168 and 163, with standard deviations of 13, 14 and 12, respectively. The significance level was set at 0.05, and the desired power of the test was established at 90%. Using PASS software version 15.0, the calculated sample size per group was determined to be 42 individuals, amounting to a total of 126 participants for the three groups combined. To account for a potential dropout rate of 10%, the total sample size was adjusted to 141 participants (*n* = 141). This adjustment ensures an adequate sample size to maintain the statistical power of the study despite possible participant attrition.

This investigation is a single-center, prospective, randomized, double-blind controlled trial, which has been approved by the Ethics Committee of the Affiliated Hospital of Xuzhou Medical University (XYFY2023-KL148-01). A total of 141 patients undergoing elective laparoscopic sleeve gastrectomy in the Department of Bariatrics and Metabolic Surgery, Affiliated Hospital of Xuzhou Medical University from June to October 2023 were selected. Inclusion criteria: patients undergoing laparoscopic sleeve gastrectomy; informed consent, voluntary participation in this experiment; ASA grade II–III; 35 kg/m^2^ ≤ BMI ≤ 50 kg/m^2^; aged 18–65 years old, normal communication. Exclusion criteria: acupoint injection contraindications (physical weakness, frequent fainting history, severe local skin infection of acupoint injection, etc.); patients allergic to anisodamine; preoperative blood gas PaCO_2_ > 50 mmHg; patients with previous history of upper abdominal surgery; patients with central nervous system or mental illness and preoperative use of mental related drugs; those with severe heart, liver, and kidney dysfunction; those who refuse to sign the informed consent; participate in other clinical trials. Rejecting criteria: surgical cancelation; secondary assessment of intubation conditions before tracheal intubation; the operation time was more than 5 h; the intraoperative blood loss was more than 2000 ml; patients admitted to ICU after operation; those who cannot cooperate with the evaluation after operation. According to the random number table method, they were divided into three groups (*n* = 47): the control group (group C), the normal saline group (group S) and the anisodamine group (group A). The patient’s assigned number was placed in an opaque, airtight bag, which was opened by an acupuncturist who was not involved in this surgery or data collection, and grouped accordingly to the grouping inside the envelope. The members of the surgery, anesthesiologists, data collectors, and statisticians involved in this study were unaware of the grouping.

### Procedures

Preoperative protocols entailed an 8-h fast and abstention from any preoperative medication. Upon entering the operating room, peripheral venous access was established, and routine monitoring of electrocardiography, non-invasive blood pressure, mean arterial pressure (MAP), heart rate (HR) and peripheral capillary oxygen saturation was initiated. The ST36 acupoint, located 3 in. below the patella’s inferior border and lateral to the anterior tibial crest, was identified. The point was marked following patient confirmation of tenderness and pain upon palpation. Invasive blood pressure monitoring was facilitated through radial artery catheterization under local anesthesia, utilizing heparin for anticoagulation and enabling blood sample collection. Anesthesia induction was achieved using a combination of midazolam (0.05 mg/kg), sufentanil (0.5 μg/kg), etomidate (0.3 mg/kg), rocuronium (0.6 mg/kg) and dexamethasone (10 mg). Post-intubation, mechanical ventilation was initiated under direct laryngoscopic guidance, employing a pressure control-volume guaranteed ventilation mode with a tidal volume set between 6 and 8 ml/kg and an inspiratory-expiratory ratio of 1:2. The oxygen flow rate was maintained at 2 L/min with an inspired oxygen concentration of 60%, and the respiratory rate was adjusted to sustain the arterial blood carbon dioxide partial pressure within the range of 35–45 cmH_2_O. Bilateral transversus abdominis plane blocks were executed under ultrasound guidance, employing 0.375% ropivacaine at a volume of 40 ml. Acupuncture point injections were administered after induction of general anesthesia. Group C was not injected with any fluid. Group S received bilateral ST36 acupoint injections of 1 ml saline each, following standard skin disinfection. The procedure entailed rapid vertical insertion of a sterile syringe needle into the subcutaneous tissue, followed by slow rotation to a depth of approximately 2 in. After ensuring no blood return, the solution was injected, and the syringe was left in place for 30 s post-injection before withdrawal. Similarly, group A received bilateral injections at the ST36 acupoint, utilizing anisodamine (Batch No. H33021707, Hangzhou Minsheng Pharmaceutical Co., Ltd.) in a sodium chloride solution of 5 mg/ml, following the identical method as group S. These acupoint injections were consistently performed by the same senior anesthesiologist proficient in the technique.

Anesthesia was maintained with a continuous intravenous infusion of propofol at a rate of 4–12 mg/kg/h and remifentanil at 0.2–1 μg/kg/min, complemented by the inhalation of 1% sevoflurane. Cis-atracurium was administered as needed based on muscle relaxation monitoring. Intraoperative fluid management involved the administration of sodium bicarbonate Ringer’s solution and hydroxyethyl starch at a volume ratio of 2:1. Hemodynamic stability was carefully managed; if the HR dropped below 40 beats/min or exceeded 100 beats/min, 0.5 mg of atropine or 10 mg of esmolol were administered intravenously, respectively. A MAP increase of more than 20% from the baseline was managed by deepening anesthesia or administering 10 mg of urapidil intravenously. Conversely, a MAP decrease of more than 20% from baseline was addressed by accelerating colloid infusion or administering 5 mg of ephedrine or 40–80 μg of phenylephrine intravenously. Sevoflurane was discontinued 20 min before the conclusion of surgery, and 50 mg of flurbiprofen axetil and 12.5 mg of dolasetron were administered intravenously. Propofol and remifentanil were maintained until the end of surgery. Upon achieving a muscle relaxation ratio of greater than 0.9, as indicated by four string stimulations, the tracheal tube was removed. The patients were then provided with oxygen via a post-anesthesia care unit (PACU) mask and transferred back to the ward once they reach the Aldrete score more than 9 points. For postoperative analgesia, a regimen was implemented whereby 5 mg of dezocine was administered whenever the NRS pain score reached or exceeded 4 points.

### Outcome Measures

Baseline data was meticulously gathered for the study, including QoR-40 scores recorded on D0, D1, D3 and D7. Additionally, the intraoperative usage rate of vasoactive drugs was noted. Post-extubation assessments at 24 h included the NRS pain score and analgesic drug use, as well as the NRS score for PONV, with 0 indicating no nausea and vomiting and 10 indicating severe nausea and vomiting. Recovery milestones such as the first time the patient was able to ambulate after the operation and the time to the first anal exhaust were also recorded. Peripheral blood samples were collected at 24-h post-operation for the evaluation of plasma levels of various biomarkers. These included SP, β-EP, MTL and GAS, all of which were measured using ELISA. All observational indicators were collected by physicians who were blinded to the group assignments of the patients to ensure the integrity and unbiased nature of the data collected.

### Statistical Analysis

Data analysis was conducted using SPSS version 26.0 software. Normally distributed continuous variables were expressed as mean ± standard deviation (SD). For between-group comparisons, one-way analysis of variance (ANOVA) was employed, while repeated measures ANOVA was utilized for within-group comparisons. Categorical data were analyzed using the *χ*^2^ test or Fisher’s exact test as appropriate. A *p*-value of less than 0.05 was considered indicative of statistical significance.

## Results

**Fig. 1:**
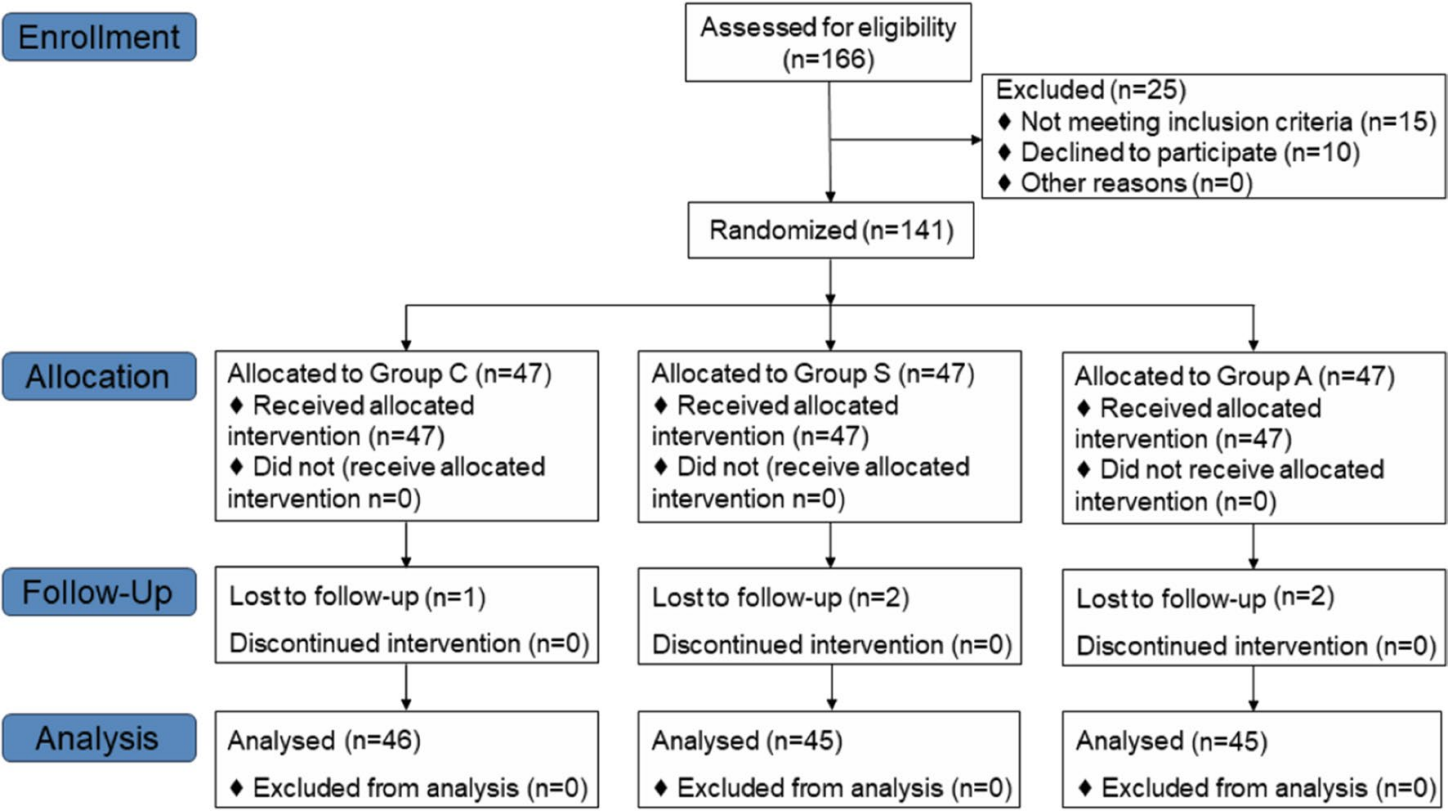
CONSORT diagram

In the current investigation, an initial cohort of 141 patients was considered for inclusion. However, 5 patients were subsequently excluded from the study due to various reasons: 2 cases were removed due to surgical cancelations, 1 patient was admitted to the ICU following postoperative extubation complications, and 2 patients were unable to cooperate with the postoperative evaluation protocols. Consequently, the statistical analysis was conducted on a final sample of 136 patients, with the distribution as follows: 46 in the control group (group C), 45 in the saline group (group S) and 45 in the anisodamine group (group A). The flow of participant inclusion and exclusion is illustrated in Fig. 1. There was no significant difference in general data among the three groups (*p* > 0.05) (Table 1).

**Table 1:** Baseline characteristics of study participants

	Group C (*n* = 46)	Group S (*n* = 45)	Group A (*n* = 45)
Age, years, mean ± SD	32.15 ± 8.11	29.53 ± 7.10	31.90 ± 6.54
Patient sex, M/F	13/33	12/33	12/33
ASA classification, II/III	23/23	22/22	19/26
BMI, kg/m^2^, mean ± SD	39.75 ± 4.42	40.79 ± 4.75	41.12 ± 4.53
Smoking, *n*(%)	11(23.9)	18(40.0)	14(31.1)
Diseases of the patients at admission, *n*(%)			
Hypertension	16(34.8)	7(15.6)	11(24.4)
Diabetes mellitus	19(41.3)	18(40.0)	17(37.8)
OSA diagnostic	17(37.0)	20(44.4)	22(48.9)
Surgery time, min, mean ± SD	95.28 ± 10.44	91.44 ± 7.12	91.78 ± 8.99
Intraoperative propofol used, mg, mean ± SD	275.54 ± 16.02	276.56 ± 12.15	276.44 ± 17.60
Intraoperative remifentanil dosage, mg, mean ± SD	1.99 ± 0.20	1.96 ± 0.26	1.94 ± 0.17
Intraoperative vasoactive drugs, *n*(%)			
Phenylephrine	34.8	28.9	35.6
Ephedrine	10.9	8.9	8.9
Atropine	2.2	2.2	4.4
Urapidil	21.7	13.3	17.8
Esmolol	10.9	8.9	4.4

*ASA*, American Society of Anesthesiologists; *BMI*, Body Mass Index; *OSA*, obstructive sleep apnoea

There were statistically significant differences in QoR-40 scores, emotional status, and pain scores among the three groups at each time point (*p* < 0.05). Compared with D0, three groups exhibited a reduction in QoR-40 scores, emotional status, physical independence and pain scores on D1; experienced a decrease in QoR-40 scores and pain scores on D3 (*p* < 0.05). Both group S and group C showed decreases in physical comfort extra during D1-3, and demonstrated in QoR-40 scores on D7(*p* < 0.05). Group C showed a reduction in pain scores on D7 (*p* < 0.05). When comparing with group C at the same points, group A exhibited an increase in QoR-40 scores and physical comfort scores from D1-3, and an increase in pain scores from D1-7; group S showed an increase in QoR-40 scores and pain scores on D1 (*p* < 0.05). Comparing with group S at the same points, group A had an increased QoR-40 scores on D1, and increased pain scores from D1-3 (*p* < 0.05) (Table 2).

**Table 2:** Total and dimensional QoR-40 scores of the participants

QoR-40	Group C(*n* = 46)	Group S(*n* = 45)	Group A(*n* = 45)	*p*-value
D0				
Physical comfort	53.6 ± 3.3	53.7 ± 3.2	53.6 ± 3.1	0.986
Emotional status	40.4 ± 2.7	40.8 ± 2.7	40.6 ± 3.0	0.787
Physical independence	23.3 ± 1.4	23.3 ± 1.4	23.2 ± 1.4	0.869
Psychological support	33.6 ± 1.3	33.6 ± 1.2	33.6 ± 1.3	0.998
Pain	33.7 ± 1.6	33.4 ± 2.1	33.4 ± 1.5	0.739
Global QoR-40	184.6 ± 6.9	184.8 ± 7.0	184.6 ± 6.2	0.984
D1				
Physical comfort	47.7 ± 5.0	49.6 ± 5.1	51.7 ± 5.6^*^	0.002
Emotional status	36.4 ± 3.9	36.1 ± 3.8	37.8 ± 3.3	0.063
Physical independence	18.8 ± 3.1	19.7 ± 3.4	20.4 ± 3.3	0.058
Psychological support	33.1 ± 1.5	33.2 ± 2.0	33.4 ± 1.2	0.765
Pain	27.6 ± 2.1	29.4 ± 3.1^*^	30.8 ± 2.4^*#^	< 0.001
Global QoR-40	163.4 ± 8.8	168.2 ± 9.0^*^	174.2 ± 9.3^*#^	< 0.001
D3				
Physical comfort	49.8 ± 5.2	51.4 ± 4.7	52.3 ± 3.7^*^	0.03
Emotional status	38.9 ± 3.4	39.3 ± 3.6	39.3 ± 3.7	0.815
Physical independence	22.5 ± 1.7	22.5 ± 2.0	22.4 ± 2.2	0.999
Psychological support	33.2 ± 1.3	33.2 ± 1.6	33.2 ± 1.6	0.977
Pain	30.1 ± 2.4	30.8 ± 2.0	32.2 ± 2.0^*#^	< 0.001
Global QoR-40	174.2 ± 8.2	177.1 ± 7.8	179.3 ± 8.1^*^	0.011
D7				
Physical comfort	53.0 ± 4.6	53.1 ± 4.0	53.1 ± 3.9	0.973
Emotional status	40.9 ± 2.4	40.7 ± 2.9	40.8 ± 3.0	0.941
Physical independence	23.2 ± 1.4	23.1 ± 1.7	23.1 ± 1.9	0.972
Psychological support	33.5 ± 1.3	33.4 ± 1.6	33.5 ± 2.1	0.942
Pain	32.3 ± 1.8	32.9 ± 1.3	33.3 ± 1.2^*^	0.011
Global QoR-40	182.8 ± 7.1	183.2 ± 7.1	183.8 ± 7.3	0.801

Data are presented as mean ± SD. ^*^p < 0.05 vs. group C; ^#^*p* < 0.05 vs. group S

**Fig. 2:**
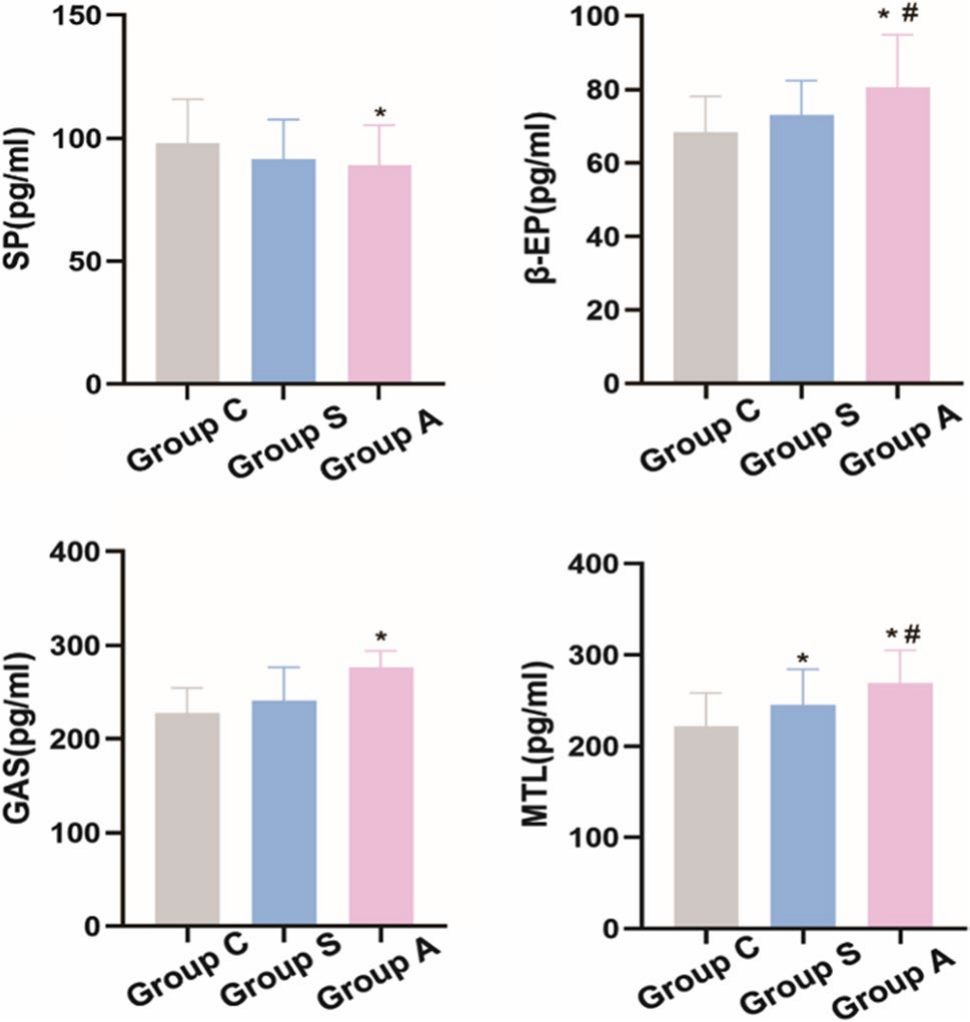
Comparison of serological markers at 24 h after operation among the three groups. Group C, the control group; Group S, the normal saline group; Group A, the anisodamine group; ^*^p < 0.05 vs. group C; ^#^*p* < 0.05 vs. group S

At 24-h post-operation, significant differences were observed in the plasma levels of SP, β-EP, MTL and GAS among the three groups (*p* < 0.05). Regarding pain markers, the SP level in group A was lower than in group C, while the β-EP level in group A was higher compared to both group S and group C. In the context of gastrointestinal hormones, the MTL level in group A exceeded those in both group S and group C, with group S also showing higher MTL levels than group C. Additionally, the GAS level in group A was higher than in group C. These differential biomarker levels among the groups are graphically represented in Fig. 2.

Across the three study groups, no statistically significant differences were noted in several key postoperative conditions: the time to extubation, duration of stay in PACU, incidence of adverse events and time to first ambulation (*p* > 0.05). The NRS pain scores 24-h post-operation exhibited significant differences among the three groups (*p* < 0.05). Specifically, group A reported lower pain scores compared to both group S (*p* < 0.05) and group C (*p* < 0.05), and group S also showed lower pain scores than group C (*p* < 0.05). Additionally, the consumption of dezocine on the first day post-operation in group A was significantly less than that in group C (*p* < 0.05). In terms of PONV, group A exhibited lower scores compared to group C (*p* < 0.05). Furthermore, the time to the first anal exhaust in group A was shorter compared to that in group C (*p* < 0.05) (Table 3).

**Table 3:** Postoperative conditions of participants

	Group C (*n* = 46)	Group S (*n* = 45)	Group A (*n* = 45)	*p*-value
Time to extubation, min	8.00 ± 2.28	8.80 ± 1.95	8.93 ± 2.26	0.088
PACU residence time, min	30.13 ± 5.40	30.69 ± 5.26	28.60 ± 6.10	0.194
Adverse events, hiccough/respiratorydepression/dizziness/urinary retention/postoperative hemorrhage	7(1/0/6/0/0)	7(1/1/5/0/0)	8(2/0/6/0/0)	0.937
NRS pain score was recorded 24 h after operation, score	5.65 ± 2.15	4.33 ± 2.12^*^	3.22 ± 2.10^*#^	< 0.001
Dezocine dose during the first postoperative day, mg	14.78 ± 6.41	12.22 ± 5.06	10.77 ± 6.99^*^	0.009
PONV score at 24 h after operation, score	5.70 ± 2.61	4.31 ± 2.95	3.73 ± 2.93^*^	0.004
Time to first out-of-bed, h	15.05 ± 4.19	14.53 ± 4.45	13.37 ± 4.32	0.167
Time to firstanal exhaust, h	27.62 ± 7.89	25.08 ± 7.27	22.62 ± 7.97^*^	0.010

Data are presented as mean ± SD. ^*^p < 0.05 vs. group C; ^#^*p* < 0.05 vs. group S

## Discussion

The aim of this study was to investigate the effect and impact of anisodamine injection at the ST36 acupoint on the quality of postoperative early recovery in patients undergoing laparoscopic sleeve gastrectomy. Surprisingly, we observed a significant beneficial effect of anisodamine injection at the ST36 acupoint in postoperative quality recovery indicators such as QoR-40 scores and pain. In addition, serological experiments also revealed significant changes in β-EP and MTL at 24 h postoperatively in patients with anisodamine injected at ST36 acupoint. To our knowledge, this is the first study on the effect of anisodamine injection at the ST36 acupoint on the quality of postoperative early recovery in patients undergoing bariatric surgery.

Patients undergoing laparoscopic sleeve gastrectomy are prone to adverse reactions such as pain and abdominal distension during the perioperative period, which seriously affects the early postoperative rehabilitation of patients [11]. As a tool for measuring clinical intervention, the QoR-40 scale created by MYLES et al. [12] can truly, comprehensively, multi-dimensionally, and effectively evaluate the impact of various factors on the quality of early postoperative recovery. An increase of more than 6.3 points in QoR-40 scores is indicative of an improvement in patient recovery [13]. This study’s findings highlight a statistically significant difference in QoR-40 scores among the three groups within the first three days post-surgery. Compared to group C, stimulation via ST36 acupoint injection was observed to enhance QoR-40 scores on the first postoperative day, with anisodamine acupoint injection further amplifying this improvement beyond the 6.3-point threshold. Notably, only in group A was there no significant difference in QoR-40 scores between D0 and D7. These results suggest that ST36 acupoint injection not only improves the quality of postoperative recovery but also that the pharmacological effect of anisodamine enhances this therapeutic impact. Interestingly, another study found that ST36 acupoint injection without or with anisodamine had no effect on the early recovery in the hospital and quality of life after discharge [10]. The difference in the results of our analyses may be due to the inconsistency in the gender of the patients involved in the study. We included both men and women in our study, and they explored only female patients. In traditional Chinese medicine, the ST36 point is known for its role in tonifying Qi, nourishing blood and ameliorating weakness [14]. Various treatment modalities, such as acupoint pressing [15], acupuncture [16] and drug injection [17], have been extensively researched and employed, underscoring the clinical relevance and utility of this approach in enhancing postoperative recovery quality.

Acupoint injection, a form of intramuscular injection, leverages the dual benefits of acupuncture point stimulation and the therapeutic effects of drugs absorbed at these points to modulate the function of zang-fu organs, guided by the principles of meridians and collaterals [18]. Anisodamine, recognized as a classic M-cholinergic receptor blocker, exhibits a multifaceted pharmacological profile, including analgesic and anti-inflammatory properties, relaxation of gastrointestinal smooth muscles, and inhibition of glandular secretions. Additionally, it can indirectly mitigate vasospasm and enhance microcirculation by antagonizing α-adrenergic receptors [19]. The combination of mechanical stimulation and pharmacological action in acupoint injection allows for prolonged needle sensation, characterized by rapid onset, minimal side effects, significant therapeutic effectiveness, and reduced drug dependence [20]. The synergistic impact of ST36 acupoint injection with anisodamine on QoR-40 scores may be attributed to the interaction among various postoperative recovery factors such as pain and gastrointestinal motility, because the adverse effects of physical comfort and emotional state scores can be caused by abdominal pain, PONV, bloating and reflux.

SP, a polypeptide, plays a crucial role in pain conduction. It is released at central nerve terminals and contributes to pain transmission by promoting the secretion and release of glutamate and other substances [21]. β-EP is an endogenous morphine biochemical synthetic hormone with endogenous analgesic effect [22]. The levels of SP and β-EP in peripheral blood serve as valuable indicators for assessing patient pain [23]. Acupoint injection has been recognized for its ability to stimulate the production of vascular endothelial growth factor and transforming growth factor β1, thereby enhancing angiogenesis and improving blood flow. This technique has been effectively used in the treatment of various painful conditions [24]. Anisodamine, as an anticholinergic agent, can alleviate pain by relaxing smooth muscles and easing microvascular spasms [25]. In this study, the postoperative SP level in group A was lower compared to group C, and the β-EP levels in groups A and S were higher than in group C. Additionally, the requirement for supplemental analgesics (dezocine) was significantly lower in group A compared to group C. The results of NRS pain score at 24 h after operation showed that acupoint injection of anisodamine at ST36 could significantly reduce the pain score. However, a reduction of 1.5 points or more is often considered clinically significant for pain relief. Hence, while acupoint injection alone may not be sufficient to achieve clinically significant short-term postoperative pain relief in bariatric surgery patients, the addition of anisodamine achieved not only statistical significance but also clinical relevance in pain score reduction.

Gastrointestinal motility disorders are frequent physiological and pathological changes observed after bariatric surgery [26]. MTL and GAS are key indicators of gastrointestinal activity and are instrumental in assessing postoperative gastrointestinal recovery [27]. ST36 acupoint injection can achieve the effect of adjuvant therapy by promoting gastrointestinal motility, treating intestinal paralysis, regulating gastrointestinal hormones, increasing intestinal beneficial bacteria, and enhancing immunity. It can be used clinically to treat stomach pain, abdominal distension and PONV [28]. Anisodamine, known for its anticholinergic properties, can relax gastrointestinal smooth muscles and alleviate gastrointestinal spasms, thereby reducing intestinal tension. It also relaxes vascular smooth muscle, which improves blood supply and can contribute to the reduction of PONV incidence [29]. This dual effect of acupoint injection and anisodamine on gastrointestinal motility and function can significantly aid in the postoperative recovery process, especially following bariatric surgery. This study showed that the nausea and vomiting scores of patients in group A were significantly reduced at 24 h after operation, suggesting the potential advantages of ST36 acupoint injection of anisodamine in preventing PONV. In addition, we found that the combined use of anisodamine on the basis of ST36 acupoint injection could increase the level of MTL compared with the control group. Concurrently, the GAS levels in group A were higher than in group C, which suggests improved gastrointestinal function. Although there was no significant difference in the initial time of ambulation among the three groups, the time to the first anal exhaust in patients receiving ST36 acupoint injection with anisodamine was noticeably shorter. This discrepancy may be attributed to adherence to postoperative care guidelines, which typically advise patients to ambulate as early as 6 h after surgery. Consequently, the reliability of data regarding the initial post-surgery ambulation time may be compromised. However, the time to the first anal exhaust is a more precise measure, and in conjunction with the MTL findings, it corroborates the positive influence of ST36 acupoint injection with anisodamine on the early recovery of gastrointestinal function in bariatric surgery patients.

Indeed, this study is not without its limitations. Firstly, the collection of clinical data was limited to the first 7 days after surgery, without extending into a long-term follow-up. This restricts our understanding of the patients' long-term prognosis and the potential for chronic postoperative pain, which are critical aspects of comprehensive patient care. Secondly, as a single-center trial, the sample size of this study was relatively small. Therefore, a multi-center, prospective clinical trial should be further implemented to evaluate the performance and robustness of anisodamine injection at the ST36 acupoint with a larger sample size and sample diversity. Such expanded research would provide a more robust and generalizable understanding of the efficacy and implications of the treatments evaluated in this study.

## Conclusion

Our study confirmed that anisodamine given by ST36 acupoint injection significantly promoted the recovery of gastrointestinal function in patients undergoing laparoscopic sleeve gastrectomy, reduced the incidence of PONV, and effectively reduced postoperative pain, thereby greatly improving the quality of postoperative early recovery, and these data suggested that it has the potential to be a valuable therapeutic measure in bariatric surgery.

## Data Availability

All data will be made available on request to the corresponding authors.

## Abbreviations

*β-EP*: β-Endorphin
*GAS*: Gastrin
*HR*: Heart rate
*MAP*: Mean arterial pressure
*MTL*: Motilin
*NRS*: Numerical rating scale
*PACU*: Post-anesthesia care unit
*PONV*: Postoperative nausea and vomiting
*QoR-40*: Quality of recovery-40 questionnaire
*SP*: Substance P
*ST36*: Zusanli
